# A radiographic model predicting the status of the anterior cruciate ligament in varus knee with osteoarthritis

**DOI:** 10.1186/s12891-022-05568-3

**Published:** 2022-06-22

**Authors:** Changquan Liu, Juncheng Ge, Cheng Huang, Weiguo Wang, Qidong Zhang, Wanshou Guo

**Affiliations:** 1 Graduate School of Peking Union Medical College and Chinese Academy of Medical Sciences, Beijing, China; 2 Department of Orthopaedic Surgery, China-Japan Friendship Hospital, Beijing, China; 3 Department of Orthopaedic Surgery, Peking University China-Japan Friendship School of Clinical Medicine, Beijing, China

**Keywords:** Uni-compartmental knee arthroplasty, Knee Osteoarthritis, Anterior cruciate ligament -deficient, ACLD, Predictive factor, Predictive model, X-rays

## Abstract

**Purpose:**

The study aims to investigate the accuracy of different radiographic signs for predicting functional deficiency of anterior cruciate ligament (ACL) and test whether the prediction model constructed by integrating multiple radiographic signs can improve the predictive ability.

**Methods:**

A total number of 122 patients from January 1, 2018, to September 1, 2021, were enrolled in this study. Among them, 96 patients were classified as the ACL-functional (ACLF) group, while 26 patients as the ACL-deficient (ACLD) group after the assessment of magnetic resonance imaging (MRI) and the Lachman’s test. Radiographic measurements, including the maximum wear point of the proximal tibia% (MWPPT%), tibial spine sign (TSS), coronal tibiofemoral subluxation (CTFS), hip–knee–ankle angle (HKA), mechanical proximal tibial angle (mPTA), mechanical lateral distal femoral angle (mLDFA) and posterior tibial slope (PTS) were measured using X-rays and compared between ACLF and ACLD group using univariate analysis. Significant variables (*p* < 0.05) in univariate analysis were further analyzed using multiple logistic regression analysis and a logistic regression model was also constructed by multivariable regression with generalized estimating models. Receiver-operating-characteristic (ROC) curve and area under the curve (AUC) were used to determine the cut-off value and the diagnostic accuracy of radiographic measurements and the logistic regression model.

**Results:**

MWPPT% (odds ratio (OR) = 1.383, 95% confidence interval (CI) = 1.193–1.603, *p* < 0.001), HKA (OR = 1.326, 95%CI = 1.051–1.673, *p* = 0.017) and PTS (OR = 1.981, 95%CI = 1.207–3.253, *p* = 0.007) were shown as predictive indicators of ACLD, while age, sex, side, TSS, CTFS, mPTA and mLDFA were not. A predictive model (risk score = -27.147 + [0.342*MWPPT%] + [0.282*HKA] + [0.684*PTS]) of ACLD using the three significant imaging indicators was constructed through multiple logistic regression analysis. The cut-off values of MWPPT%, HKA, PTS and the predictive model were 52.4% (sensitivity:92.3%; specificity:83.3%), 8.5° (sensitivity: 61.5%; specificity: 77.1%), 9.6° (sensitivity: 69.2%; specificity: 78.2%) and 0.1 (sensitivity: 96.2%; specificity: 79.2%) with the AUC (95%CI) values of 0.906 (0.829–0.983), 0.703 (0.574–0.832), 0.740 (0.621–0.860) and 0.949 (0.912–0.986) in the ROC curve.

**Conclusion:**

MWPPT% (> 52.4%), PTS (> 9.6°), and HKA (> 8.5°) were found to be predictive factors for ACLD, and MWPPT% had the highest sensitivity of the three factors. Therefore, MWPPT% can be used as a screening tool, while the model can be used as a diagnostic tool.

## Introduction

Knee osteoarthritis (OA), one of the most common orthopedic diseases, often leads to pain, limited range of motion, and joint deformation [1, 2]. The incidence of the disease is increasing, thus bringing a huge burden to the medical health system with the aging of the population [2–4].

Uni-compartment knee arthroplasty (UKA) is a successful and reliable option for minimally invasive treatment of anteromedial osteoarthritis (AMOA), which can significantly improve functional recovery, kinematic alignment, and quality of life for patients [5–7]. However, strict compliance with surgical indications is a key factor in the success of UKA, especially the need to evaluate the functional integrity of the anterior cruciate ligament (ACL) before surgery [8, 9]. Functionally insufficient ACL often leads to failure of UKA surgery, and an ACL with functional integrity is a prerequisite for a successful medial UKA surgery [10–13]. In addition, bicruciate-retaining total knee arthroplasty (TKA) needs an ACL with functional integrity as well [14].

According to previous literature, there have been several radiographic signs related to the status of ACL, including the tibial wear pattern on lateral radiographs [15, 16], coronal tibiofemoral subluxation (CTFS) on anterior–posterior (AP) radiographs [17, 18], posterior tibial slope (PTS) on lateral radiographs [19, 20], and so on. However, the diagnostic accuracy of those radiographic signs varied in different pieces of literature, and related literature lacked the calculation of the cut-off value of those radiographic signs. In addition, there was no report on the prediction model of functional ACL deficiency that integrated multiple radiographic signs.

The purposes of this study were to investigate (1) the relationship between different radiographic signs and the functional status of ACL; (2) the accuracy of different radiographic signs for predicting functional deficiency of ACL; (3) whether the predictive model constructed by integrating multiple radiographic signs can improve the predictive ability. We hypothesized that these radiographic signs (the maximum wear point of the proximal tibia% (MWPPT%), PTS, and HKA) were predictive factors for the functional deficiency of ACL and a predictive model constructed by integrating those radiographic signs can improve the predictive ability.

## Methods

**Fig. 1:**
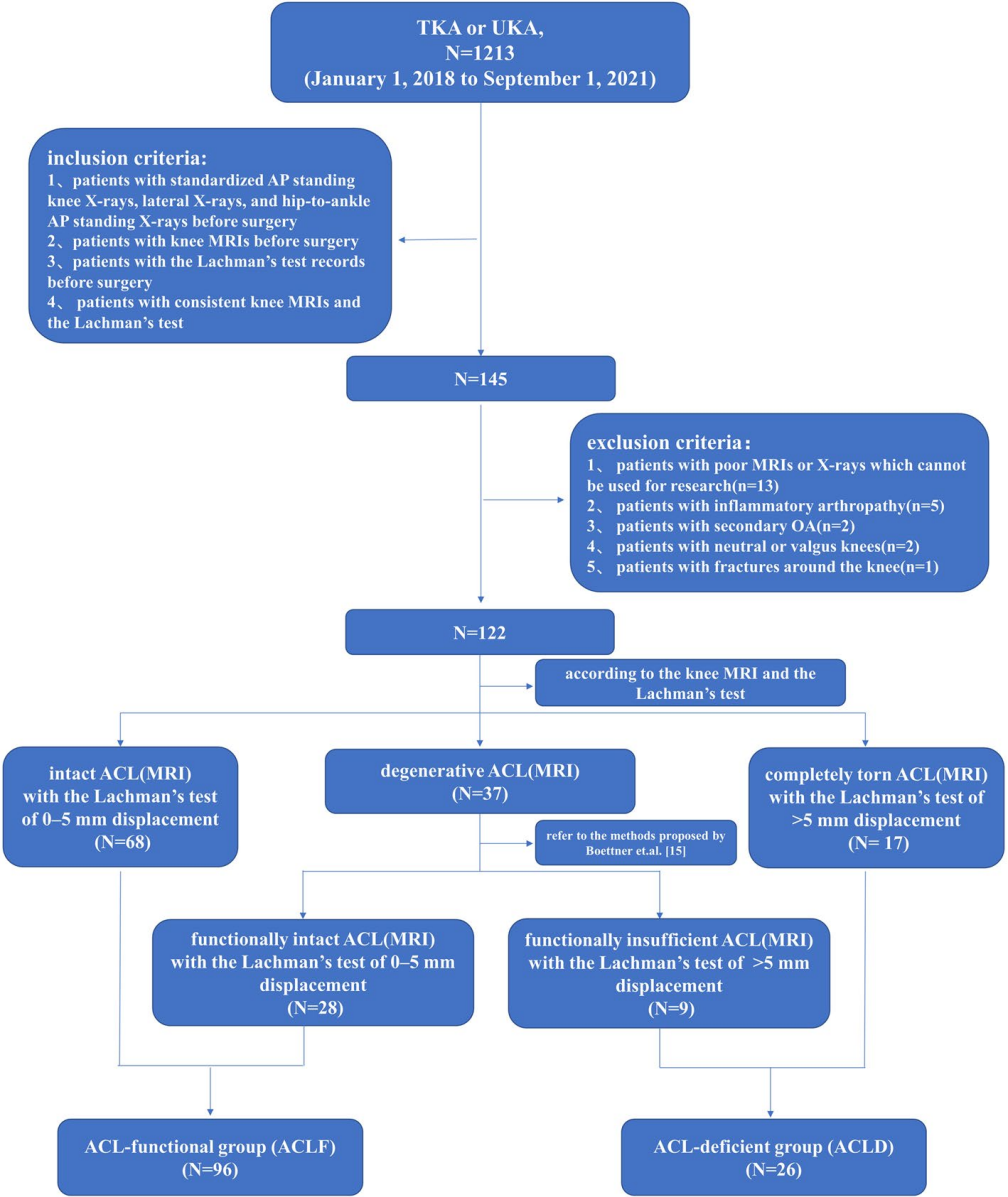
Flow chart of the study

This retrospective study was conducted using patients from January 1, 2018, to September 1, 2021, in China-Japan Friendship Hospital. The inclusion criteria were as follows: (1) patients undergoing uni-compartmental knee arthroplasty or total knee arthroplasty for varus knee with osteoarthritis; (2) patients with knee magnetic resonance imaging (MRI); (3) patients with standardized AP standing knee X-rays, lateral X-rays, and hip-to-ankle AP standing X-rays before knee surgery; (4) patients with the Lachman’s test records before surgery; (5) patients with consistent knee MRI evaluation and the Lachman’s test. The exclusion criteria were as follows:(1) patients with poor MRI or X-rays which can’t be used for research (*n* = 13); (2) patients with inflammatory arthropathy (*n* = 5); (3) patients with secondary OA (*n* = 2); (4) patients with neutral or valgus knees (*n* = 2); (5) patients with fractures around the knee (*n* = 1). A total number of 122 patients were enrolled in this study. The flow chart was shown in Fig. 1. All patients were evaluated preoperatively to assess ACL integrity with the Lachman’s test (an anterior force was applied to the tibia while the knee was flexed at 20° to 30°). The Lachman’s test was graded as 0–5 mm displacement and > 5 mm displacement, and we considered > 5 mm displacement as indicating ACL instability [21]. The study was approved by the institutional review board of the China-Japan Friendship Hospital (approval number 2020–50-k28).

### MRI assessment

All subjects underwent high-quality preoperative MRI using a GE Signa 1.5 T magnetic resonance imaging system (GE Company, USA). Patients were positioned in supine with the knee extended. The special coil for the knee joint was selected to perform routine serial scanning on the cross-section, sagittal plane, and coronal plane. The scanning parameters were SE-T1WI (TR = 540 ms, TE = 13 ms, 5 mm, FOV17 × 17) and FSE-T2WI (TR = 4500 ms, TE = 39.8 ms, 5 mm, FOV 17 × 17). The MRI was used to assess the status of ACL, graded as intact, with degenerative changes (including scarring, thinning, mucoid degeneration, ganglion formation, or partial tears) and completely torn [22, 23]. Based on the methods proposed by Boettner et.al. [15], the degenerative status can be further divided into functionally insufficient (< 14% posterior intact cartilage of the medial compartment) and functionally intact (> 14% posterior intact cartilage of the medial compartment) status. In this study, intact and degenerative (functionally intact) status (0–5 mm of displacement in the Lachman’s test at the same time) were classified as the ACL-functional (ACLF) group (functional integrity of ACL), while completely torn and degenerative (functionally insufficient) status (> 5 mm of displacement in the Lachman’s test at the same time) were regarded as the ACL-deficient (ACLD) group (functional deficiency of ACL). Of all patients, 96 patients were classified as the ACL-functional (ACLF) group, while 26 patients as the ACL-deficient (ACLD) group after the assessment of MRI and the Lachman’s test (Fig. 1). The status of ACL based on MRI was evaluated by two orthopedic surgeons using the hospital's imaging system (picture archiving and communication system, PACS), and the kappa’s coefficient was 0.915 (95%CI = 0.848 to 0.982), showing good interobserver reliability.

### Radiographic assessment

Before surgery, all patients had standardized AP standing knee X-rays, lateral X-rays, and hip-to-ankle AP standing X-rays. These radiographic signs were measured using X-rays: MWPPT%, tibial spine sign (TSS), coronal tibiofemoral subluxation (CTFS), hip–knee–ankle angle (HKA), mechanical proximal tibial angle (mPTA), mechanical lateral distal femoral angle (mLDFA) and posterior tibial slope (PTS).

**Fig. 2:**
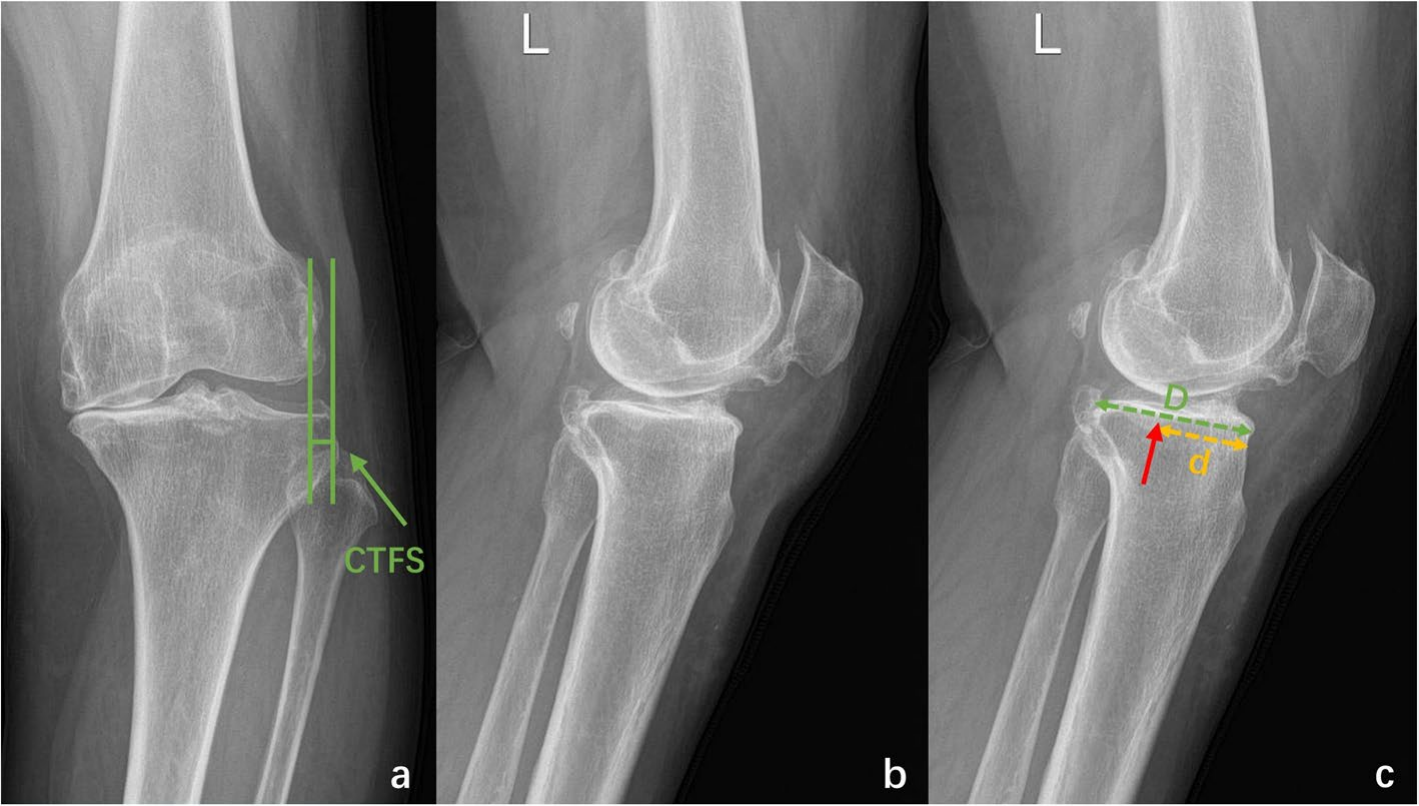
Measurement of Coronal tibiofemoral subluxation (CTFS) and the maximum wear point of the proximal tibia (MWPPT) **a-c** The anterior–posterior (AP) standing knee X-ray and lateral X-ray of an anterior cruciate ligament-deficient (ACLD) patient (**a-c**). CTFS is defined as the distance between the tangent line to the outermost joint edge of the lateral condyle of the femur and the tangent line of the lateral tibial plateau (**a**). The red arrow indicates the maximum wear point of the proximal tibia. MWPPT% is recorded as the ratio between the distance from the maximum wear point to the anterior edge of the tibia (yellow dotted line) and the length of the medial tibial plateau (green dotted line) (**c**)

The AP standing knee X-rays were used to measure CTFS and TSS. CTFS was defined as the distance between the tangent line to the outermost joint edge of the lateral condyle of the femur and the tangent line of the lateral tibial plateau [18, 24] (Fig. 2). TSS included three types on the standardized AP standing knee X-rays: type0—no contact between the lateral condyle of the femur and the lateral intercondylar spine of the tibia, type1—contact between lateral condyle of the femur and lateral intercondylar spine of the tibia, type2 – the overlap of lateral condyle of the femur and lateral intercondylar spine of the tibia [25].

**Fig. 3:**
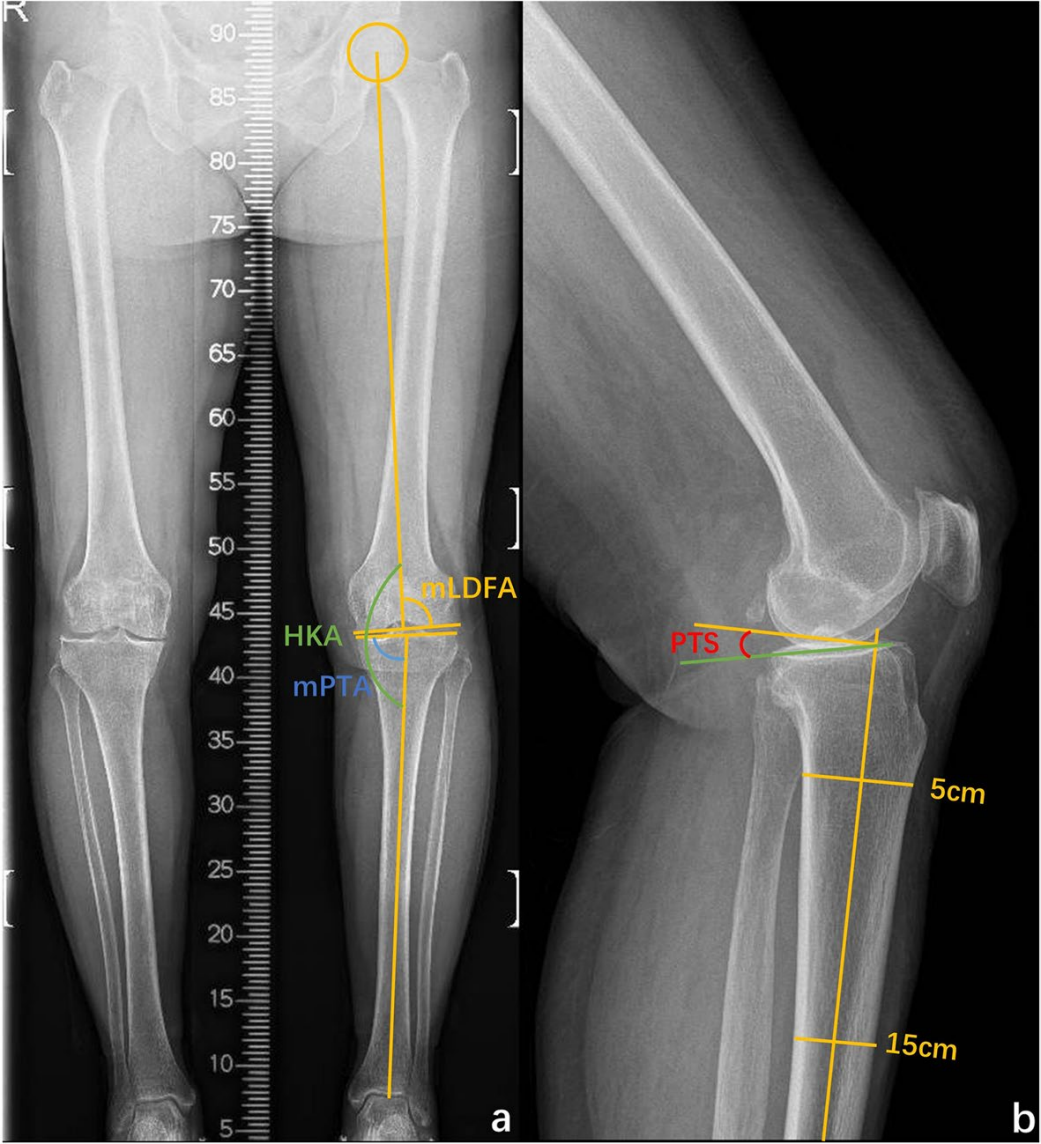
Measurement of different alignment parameters on hip-to-ankle anterior–posterior (AP) standing (**a**) and lateral (**b**) X-rays. Hip–knee–ankle angle (HKA) is the angle between the tibial mechanical axis and femoral mechanical axis, mechanical proximal tibial angle (mPTA) is the angle between the tangent of the medial and lateral tibial plateau and the mechanical axis of the tibia, and mechanical lateral distal femoral angle (mLDFA) is the angle between the tangent of the medial and lateral femoral condyle and the mechanical axis of the femur. Posterior tibial slope (PTS) is the angle between the tibial anatomical axis (a straight line connecting the midpoint of the line at 5 cm and 15 cm from the knee joint line) and tibial plateau (a line connecting the anterior and posterior points of the most proximal part of the tibial plateau)

The lateral X-rays were used to measure MWPPT% and PTS. MWPPT% was recorded as the ratio between the distance from the maximum wear point to the anterior edge of the tibia and the length of the medial tibial plateau [26, 27] (Fig. 2). PTS was the angle between the tibial anatomical axis (a straight line connecting the midpoint of the line at 5 cm and 15 cm from the knee joint line) and the tibial plateau (a line connecting the anterior and posterior points of the most proximal part of the tibial plateau) [28] (Fig. 3).

On hip-to-ankle AP standing X-rays, HKA, mPTA, and mLDFA were measured (Fig. 3). HKA (recorded as a varus angle in our study) was the angle between the tibial mechanical axis and femoral mechanical axis, mPTA was the angle between the tangent of the medial and lateral tibial plateau and the mechanical axis of the tibia, and mLDFA was the angle between the tangent of the medial and lateral femoral condyle and the mechanical axis of the femur [29, 30].

All measurements were made by two orthopedic surgeons using the hospital's imaging system (PACS). Intra-class correlation coefficient (ICC) was used for continuous variables (MWPPT%, CTFS, PTS), while kappa’s coefficient was used for nominal variables (TSS) to test the interobserver reliability.

### Statistical analysis

The continuous variables (age, MWPPT%, CTFS, HKA, mPTA, mLDFA, PTS) were presented as means and standard deviations (SD), while the categorical variables (sex, side, TSS) were given as frequencies and percentages (%).

The Shapiro–Wilk test was used to examine the normality of continuous variables. In univariate analysis between ACLF and ACLD groups, the independent-samples t-test or the Mann–Whitney U-test was used for continuous variables, while the Chi-square test or the Fisher’s exact test was used for categorical variables. Significant variables (*p* < 0.05) in univariate analysis were further analyzed using multiple logistic regression analysis to assess the predictive variables of ACLD and a predictive model of ACLD was constructed by multiple logistic regression analysis with generalized estimating models. Stepwise logistic regression analysis with backward elimination was performed according to the Akaike information criterion. Receiver operating characteristic (ROC) curves and area under the curve (AUC) were used to determine the cut-off value and the diagnostic accuracy of radiographic measurements and the logistic regression model.

Power analysis was performed in G-power (G*Power Version3.1.9, Germany) for the Mann–Whitney U test using an alpha of 0.05, a power of 80%, and an effect size of 0.5 resulting in a sample size of 106. We included a total of 122 patients in the study.

All statistical analyses were performed using SPSS24.0 (IBM, New York, USA), and a *p* < 0.05 (two-sides) was considered statistically significant.

## Results

### Subject characteristics

A total of 122 patients were enrolled in the study. The mean ± SD of age was 66.82 ± 8.40. The mean ± SD of MWPPT%, CTFS, HKA, mLDFA, mPTA, and PTS were 49.96 ± 6.41, 5.17 ± 1.47, 7.66 ± 3.81, 89.32 ± 2.16, 85.68 ± 2.08 and 8.98 ± 1.97. Of all patients, 21 patients were male and 101 patients were female; 61 patients had surgery on the left knee, while the others (61 patients) on the right knee; 76 patients were classified as type 0 of tibial spine sign, 20 patients as type 1 and 26 patients as type 2 (Table 1). All patients were divided into two groups, ACLF (*n* = 96) and ACLD (*n* = 26), and the demographic characteristics of each group were presented in Table 1. The ICCs (95%CI) of imaging indicators (MWPPT%, CTFS, and PTS) were 0.853 (0.796–0.895), 0.846 (0.787–0.890) and 0.807 (0.734–0.861), and the κ (95%CI) of imaging indicator (TSS) was 0.910 (0.841–0.979), all showing good interobserver reliability (Table 2).

**Table 1:** Basic characteristic

Variables	Total (*n* = 122)	ACLF (*n* = 96)	ACLD (*n* = 26)	*p*
Age(years)	66.82 ± 8.40	66.36 ± 8.71	68.50 ± 7.24	0.254^a^
Sex				0.560^b^
Female	101 (82.8%)	78 (81.2%)	23 (88.5%)	
Male	21 (17.2%)	18 (18.8%)	3 (11.5%)	
Side				0.658^c^
Left	61 (50.0%)	52 (54.2%)	9 (34.6%)	
Right	61 (50.0%)	44 (45.8%)	17 (65.4%)	
MWPPT%	49.96 ± 6.41	47.83 ± 4.69	57.83 ± 5.93	< 0.001^d^
TSS				0.376^b^
0	76 (62.3%)	57 (59.4%)	19 (73.1%)	
1	20 (16.4%)	16 (16.7%)	4 (15.4%)	
2	26 (21.3%)	23 (23.9%)	3 (11.5%)	
CTFS (mm)	5.17 ± 1.47	4.91 ± 1.24	6.13 ± 1.89	0.003^d^
HKA (°)	7.66 ± 3.81	6.94 ± 3.07	10.34 ± 5.03	0.003^a^
mLDFA (°)	89.32 ± 2.16	89.19 ± 2.12	89.80 ± 2.31	0.078^a^
mPTA (°)	85.68 ± 2.08	85.82 ± 2.20	85.18 ± 1.57	0.095^a^
PTS (°)	8.98 ± 1.97	8.62 ± 1.90	10.28 ± 1.73	< 0.001^d^

*ACLF* Anterior cruciate ligament-functional, *ACLD* Anterior cruciate ligament-deficient, *MWPPT%* the maximum wear point of the proximal tibia%, *TSS* Tibial spine sign, *CTFS* Coronal tibiofemoral subluxation, *HKA* Hip–knee–ankle angle, *mLDFA* mechanical lateral distal femoral angle, *mPTA* mechanical proximal tibial angle, *PTS* Posterior tibial slope

^a^ the independent-samples t-test

^b^ the Fisher’s exact test

^c^ the Chi-square test

^d^ the Mann–Whitney U-test

**Table 2:** Interobserver reliability

Variables	ICC or κ	95% CI	*p*
MWPPT%, ICC	0.853	0.796–0.895	< 0.001
TSS, κ	0.910	0.841–0.979	< 0.001
CTFS (mm), ICC	0.846	0.787–0.890	< 0.001
PTS (°), ICC	0.807	0.734–0.861	< 0.001

*ICC* Intraclass correlation coefficient, *CI* Confidence interval, *MWPPT%* the maximum wear point of the proximal tibia%, *TSS* Tibial spine sign, *CTFS* Coronal tibiofemoral subluxation, *PTS* Posterior tibial slope

### Univariate analysis

In univariate analysis, there was no significant difference in these variables (age, sex, side, TSS, mLDFA, mPTA) between the ACLF group and ACLD group, but significant differences were found in MWPPT% (ACLF: 47.83 ± 4.69 < ACLD: 57.83 ± 5.93, *p* < 0.001), CTFS (ACLF: 4.91 ± 1.24 < ACLD: 6.13 ± 1.89, *p* = 0.003), HKA (ACLF: 6.94 ± 3.07 < ACLD: 10.34 ± 5.03, *p* = 0.003) and PTS (ACLF: 8.62 ± 1.90 < ACLD: 10.28 ± 1.73, *p* < 0.001) (Table 1).

### Multiple logistic regression analysis and the predictive model construction

The significant variables (MWPPT%, CTFS, HKA and PTS) in univariate analysis were further analyzed by multiple logistic regression analysis. The three variables, including MWPPT% (OR = 1.383, 95%CI = 1.193–1.603, and *p* < 0.001), HKA (OR = 1.326, 95%CI = 1.051–1.673, and *p* = 0.017) and PTS (OR = 1.981, 95%CI = 1.207–3.253, and *p* = 0.007), were expressed as the predictive variables of ACLD in the multiple logistic regression analysis. Through multiple logistic regression analysis, a predictive model (with percentage accuracy in classification of 89.3%) was also constructed using the three significant imaging indicators: risk score = -27.147 + [0.342*MWPPT%] + [0.282*HKA] + [0.684*PTS] (Table 3).

**Table 3:** Multivariate logistic regression analysis of predictive factors for anterior cruciate ligament-deficient (ACLD)

Variables	OR	B value ± S.E	95% CI	*P*
MWPPT%	1.383	0.342 ± 0.075	1.193–1.603	< 0.001
CTFS (mm)	1.071	0.068 ± 0.306	0.588–1.951	0.824
HKA (°)	1.326	0.282 ± 0.119	1.051–1.673	0.017
PTS (°)	1.981	0.684 ± 0.253	1.207–3.253	0.007
Constant	-	-27.147 ± 5.439	-	< 0.001

*OR* Odds ratio, *CI* Confidence interval, *MWPPT%* the maximum wear point of the proximal tibia%, *CTFS* Coronal tibiofemoral subluxation, *HKA* Hip–knee–ankle angle, *PTS* Posterior tibial slope

Logistic regression model: risk score = -27.147 + [0.342*MWPPT%] + [0.282*HKA] + [0.684*PTS]

### Comparison of the ROC curves for radiographic measurements and logistic regression model

**Fig. 4:**
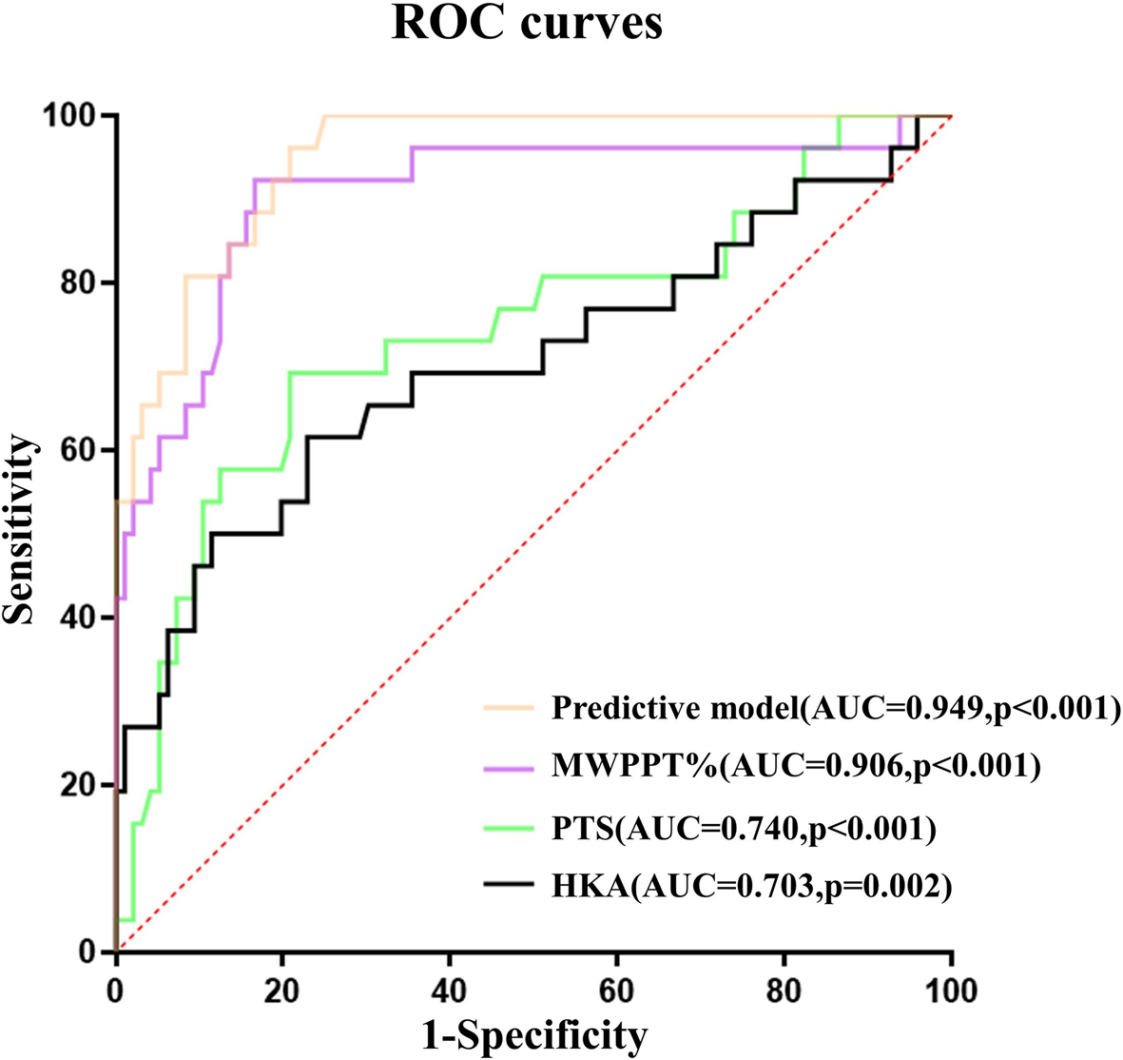
The receiver operating characteristic (ROC) curve for MWPPT%, HKA, PTS, and predictive model. The area under the curve (AUC) was 0.906(0.829–0.983) for MWPPT%, 0.703(0.574–0.832) for HKA, 0.740(0.621–0.860) for PTS and 0.949(0.912–0.986) for predictive model (95%CI). MWPPT%, the maximum wear point of the proximal tibia%; HKA, Hip–knee–ankle angle; PTS, posterior tibial slope; CI, confidence interval

In the ROC curves of significant variables (MWPPT%, HKA and PTS) in logistic regression analysis, the cut-off values were 52.4%, 8.5° and 9.6° with the AUC (95% CI) values of 0.906 (0.829–0.983), 0.703 (0.574–0.832) and 0.740 (0.621–0.860). The sensitivity of MWPPT%, HKA, and PTS were 92.3%, 61.5% and 69.2%, while the specificity of these indicators were 83.3%,77.1% and 78.2% (Table 4 and Fig. 4).

**Table 4:** Comparison of predictive factors and predictive model of anterior cruciate ligament-deficient (ACLD)

Variables	AUC (95% CI)	Youden index^b^	Cut-off value	Sensitivity%	Specificity%	*p*
MWPPT%	0.906(0.829–0.983)	0.756	52.4	92.3	83.3	< 0.001
HKA **(°)**	0.703(0.574–0.832)	0.386	8.5	61.5	77.1	0.002
PTS **(°)**	0.740(0.621–0.860)	0.484	9.6	69.2	78.2	< 0.001
Predictive model^a^	0.949(0.912–0.986)	0.754	0.1	96.2	79.2	< 0.001

*AUC* Area under the curve, *CI* Confidence interval, *MWPPT%* the maximum wear point of the proximal tibia%, *HKA* Hip–knee–ankle angle, *PTS* Posterior tibial slope

^a^ Predictive model: risk score = -27.147 + [0.342*MWPPT%] + [0.282*HKA] + [0.684*PTS]

^b^ Youden index = sensitivity + specificity—1

For the predictive model (risk score = -27.147 + [0.342*MWPPT%] + [0.282*HKA] + [0.684*PTS]), the cut-off value of the risk score was 0.1 and the AUC (95% CI) was 0.949 (0.912–0.986) in the ROC curve. In addition, the sensitivity and specificity of the model were 96.2% and 79.2% when predicting ACLD (Table 4 and Fig. 4).

## Discussion

Our research found that the three imaging indicators, including MWPPT%, HKA, and PTS, were predictors of ACLD, and the cut-off values of the three indicators were calculated with the accuracy (AUC value), sensitivity, and specificity values. We further built a prediction model that combined the above three indicators to further improve the accuracy of the prediction of ACLD. It was worth mentioning that MWPPT% had the highest sensitivity of the three indicators. Therefore, MWPPT% can be used to screen for ACLD, and the model constructed in the study can be further used as a diagnostic tool to determine the status of ACL.

As we all know, the surgical indications for medial UKA include the following two points:(1) anteromedial osteoarthritis (AMOA) confirmed using the radiological examination. (2) knee joint stability, which means medial and lateral collateral ligaments, and anterior and posterior cruciate ligament are intact [8, 9]. For patients who are preparing for UKA surgery, it is extremely important to assess the status of the patient's ACL before surgery. The insufficient function of ACL often leads to failure of UKA surgery, and an ACL with functional integrity is a prerequisite for a successful medial UKA surgery [10–12]. In addition, bicruciate-retaining TKA also needs ACL with functional integrity [14]. However, the intact ACL through preoperative physical examination can sometimes be found to be damaged or even broken during the operation, and not all patients preparing for UKA or bicruciate-retaining TKA have preoperative MRI [21, 31]. Therefore, it is meaningful to find a preoperative radiographic method with high accuracy for ACL functional status assessment.

Our study revealed that the three imaging indicators, including MWPPT%, PTS, and HKA, were predictors of ACLD. MWPPT%-The known Keyes classification used the lateral radiograph to assess the status of ACL and graded the ACL based on the tibia wear pattern on the lateral radiograph [16]. Many studies have shown that there was a correlation between the tibia wear pattern on the lateral radiograph and the status of the ACL [15, 27, 32]. When the status of ACL changed from intact ACL to functionally deficient ACL, the location of tibia wear on the lateral radiograph moved from anterior to posterior tibial plateau. However, those studies only described the phenomenon qualitatively, not quantitatively. In our study, we quantified the tibia wear pattern on the lateral radiograph using MWPPT% and we found that MWPPT% of 52.4% was the cut-off value to predict the ACLD with a sensitivity of 92.3% and a specificity of 83.3% (AUC value: 0.906) (Table 4). It was worth mentioning that this study is the first to quantify the wear of the tibia pattern on the lateral radiograph to predict ACLD using the Asian population. PTS-It has been reported in the literature that there was a correlation between PTS and ACL damage [19, 20, 33]. The larger the PTS, the more easily the ACL was damaged. However, related research was limited to the relationship between non-contact ACL deficiency and PTS in young people, not contact ACL deficiency in the elderly. Recently, in a study of elderly people undergoing UKA surgery, Plancher et.al. [20] found that patients with ACL deficiency had greater preoperative PTS than patients with intact ACL (7.6 ± 2.8° > 5.4 ± 5.9°, *p* = 0.001). In our study, we found a similar result that patients with functional ACL deficiency have larger preoperative PTS (ACLD: 10.28 ± 1.73° > ACLF 8.62 ± 1.90°, *p* < 0.001) (Table 1). The different values in the two studies might due to the variety of race and measurement methods. Further, we calculated the cut-off value (9.6°) of PTS with the corresponding sensitivity (69.2%), specificity (78.2%), and AUC (0.740), which showed that PTS was a good predictor of contact ACLD in the elderly (Table 4). HKA-Mullaji et.al. [32] and Springer et.al [18]. reported that patients with functionally deficient ACL had a greater varus angle than patients with functionally integral ACL. We had a similar result. In our research, we found that HKA in the ACLD group was larger than that in the ACLF group (ACLF: 6.94 ± 3.07 < ACLD: 10.34 ± 5.03, *p* = 0.003) (Table 1). These results might be explained by the following theory: functionally deficient ACL could lead to the relaxation of the anterior part of the knee joint, which further led to knee osteoarthritis and varus deformities [34]. Moreover, we calculated the cut-off value of HKA (8.5°) with the sensitivity and specificity of 61.5% and 77.1% when predicting ACLD (Table 4). As the varus angle was only required to be less than 15° in a surgical indication of medial UKA, more attention needed to be paid to the functional integrity of ACL for patients with varus angles greater than 8.5° preoperatively [8].

Previous studies have reported the relationship between intraoperative findings of ACL and preoperative evaluations with physical examination, radiographs, or MRI [15, 21, 35]. Waldstein et.al. [15] reported that patients with > 14% posterior intact cartilage of the medial compartment (assessed by MRI) were more likely to have a functionally intact ACL. Tao et.al. [35] found that an axial global passive anterior tibial subluxation (PATS) (> 1.2 mm) on MRI could be used as a predictive factor for a functionally deficient ACL, with the AUC, sensitivity, and specificity values of 0.897, 55% and 100%. Johnson et.al. [21] found that the preoperative Lachman test together with MRI could provide a sensitivity of 93.3% and a specificity of 99% for assessing the ACL status intraoperatively. In our study, the preoperative Lachman test together with MRI was used to determine the status of the ACL, and a predictive model of ACLD was constructed by using three radiographic indicators (MWPPT%, PTS, and HKA). It was worth mentioning that the predictive model in our study had a higher accuracy (AUC = 0.949) than the predictive index (AUC = 0.897) of Tao et.al. [35], and higher sensitivity (96.2%) than the predictive index (55.0% and 93.3%) of Tao et.al. [35] and Johnson et.al. [21]. In clinical practice, the use of a single radiographic indicator to assess the status of ACL had great uncertainty, while a model constructed using multiple indicators could greatly improve the accuracy. At present, there have been many models with multiple indicators showing high predictive accuracy in other fields [36–38]. As far as we knew, this was the first study combining multiple imaging indicators to construct an ACLD prediction model. The prediction model in our study had high accuracy (AUC = 0.949) in predicting ACLD than that of the single radiographic indicators, MWPPT% (AUC = 0.906), PTS (AUC = 0.740), and HKA (AUC = 0.703), and could be used as a diagnostic tool of ACLD. As the predictive model in our study could determine the functional status of ACL through X-rays with high accuracy (AUC = 0.949), it had certain applicable value in clinical practice, for example, determining whether further evaluation (such as MRI) was needed and whether the surgical plan should be changed.

Our study had its limitations. First, the study was a single-center, retrospective clinical study. A multi-center and prospective clinical study is needed in the future. Second, the prediction model in this study had not been verified. Therefore, the model needs to be verified later, and the result of external verification is more reliable. Third, this retrospective study lacked the intraoperative data to judge the ACL status. However, all the subjects included in the study had MRI and the Lachman’s test record, and the ACL state was judged using MRI and the Lachman’s test. Fourth, considering the rotation of patients' lower limbs and the difference in alignments, it was difficult to obtain accurate X-rays of all patients, which might affect the accuracy and repeatability of the radiographic measurements. However, the interobserver reliability of imaging indicators in this study showed good consistency in radiographic measurements.

## Conclusion

In this study, MWPPT% (> 52.4%), PTS (> 9.6°), and HKA (> 8.5°) were found to be predictive factors for ACLD and MWPPT% had the highest sensitivity of the three factors. Therefore, MWPPT% can be used as a screening tool and the model can be used as a diagnostic tool to help clinicians better judge the functional status of ACL through X-rays.

## Data Availability

The datasets used and/or analyzed during the current study are available from the corresponding author on reasonable request.

## Abbreviations

ACL: Anterior cruciate ligament
ACLF: Anterior cruciate ligament-functional
ACLD: Anterior cruciate ligament-deficient
MRI: Magnetic resonance imaging
MWPPT: Maximum wear point of the proximal tibia
TSS: Tibial spine sign
CTFS: Coronal tibiofemoral subluxation
HKA: Hip–knee–ankle angle
mPTA: Mechanical proximal tibial angle
mLDFA: Mechanical lateral distal femoral angle
PTS: Posterior tibial slope
ROC: Receiver-operating-characteristic
AUC: Area under the curve
OR: Odds ratio
CI: Confidence interval
OA: Osteoarthritis
UKA: Uni-compartment knee arthroplasty
AMOA: Anteromedial osteoarthritis
TKA: Total knee arthroplasty
AP: Anterior–posterior
PACS: Picture archiving and communication system
ICC: Intra-class correlation coefficient
SD: Standard deviations
